# The Association of *Toxoplasma gondii* with the Combination of Cardiovascular Disease, Chronic Kidney Disease, or Chronic Liver Disease: A Preliminary Study

**DOI:** 10.3390/medsci11040065

**Published:** 2023-10-03

**Authors:** Amani Babekir, Sayed Mostafa, Emmanuel Obeng-Gyasi

**Affiliations:** 1 Department of Built Environment, North Carolina A&T State University, Greensboro, NC 27411, USA; 2 Environmental Health and Disease Laboratory, North Carolina A&T State University, Greensboro, NC 27411, USA; 3 Department of Mathematics and Statistics, North Carolina A&T State University, Greensboro, NC 27411, USA

**Keywords:** *Toxoplasma gondii*, cardiovascular, kidney, liver, immune, U.S., disease

## Abstract

*Toxoplasma gondii* is a protozoan parasite widespread worldwide, with over 40 million individuals in the United States. It may infect vital organs such as the heart, kidneys, and liver, resulting in chronic infections. The main objective of this study is to investigate the association of *Toxoplasma* infection with the combination of cardiovascular disease, chronic kidney disease (CKD), or chronic liver disease (CLD). The National Health and Nutrition Examination Survey (NHANES 2009–2010) data were used, and the association of infection with chronic disease was assessed with biomarkers and indexes using statistical modeling. The percentage of participants with a combination of CLD and CKD was higher among *Toxoplasma* positive participants compared to the negative participants (2.76 vs. 1.26). Furthermore, exposure to *T. gondii* may increase the odds of cardiovascular disease, CKD, or CLD, or vice versa.

## 1. Introduction

*Toxoplasma gondii* is a parasitic protozoan that infects humans and other warm-blooded animals [1]. It is a common parasite that can cause a variety of diseases in humans, ranging from mild flu-like symptoms to more severe illnesses [2]. While toxoplasmosis is generally not a serious health problem in healthy individuals, it can cause complications in people with weakened immune systems, as well as in pregnant women and their fetuses [3]. In recent years, there has been increasing evidence linking toxoplasmosis to several chronic diseases, including cardiovascular disease, chronic kidney disease, and chronic liver disease.

Cardiovascular disease is a leading cause of death worldwide [4]. The Centers for Disease Control and Prevention indicated that one person dies every 33 s in the U.S. It is a broad term that encompasses a range of conditions affecting the heart and blood vessels, including coronary artery disease, heart failure, and stroke. There is growing evidence suggesting that toxoplasmosis may contribute to the development of cardiovascular disease [5,6]. Studies have shown that individuals infected with *T. gondii* have a higher risk of developing atherosclerosis, a condition in which fatty deposits build up in the arteries, leading to restricted blood flow and an increased risk of heart attack and stroke [7]. Additionally, toxoplasmosis may lead to chronic inflammation, which is known to contribute to the development of cardiovascular disease [8].

Chronic kidney disease (CKD) is a condition in which the kidneys gradually lose function over time [9]. It is a common condition that affects millions of people worldwide, and it can lead to a range of complications, including anemia, bone disease, and cardiovascular disease [10]. There is some evidence to suggest that toxoplasmosis may contribute to the development of CKD. Studies have shown that individuals with CKD are more likely to have antibodies to *T. gondii*, indicating previous exposure to the parasite [11]. Additionally, toxoplasmosis may contribute to the development of chronic inflammation, which is known to be a risk factor for CKD.

Chronic liver disease CLD is a condition in which the liver is damaged and cannot function properly. It can be caused by a range of factors, including alcohol abuse, viral infections, and autoimmune disorders [12]. Some studies have suggested that *T. gondii* may contribute to liver damage in individuals [13]. For example, one study found that those infected with *T. gondii* may have higher odds of nonalcoholic fatty liver disease (NAFLD) [14] with age serving as a critical factor in this association.

The cardiovascular system, hepatic system, and renal system are all critical components of human physiology, and they are intimately linked to one another [15,16]. The cardiovascular system, which includes the heart and blood vessels, is responsible for delivering oxygen and nutrients to all parts of the body [17]. The hepatic system, which includes the liver, is responsible for filtering blood, producing bile, and regulating a variety of metabolic functions [18]. The renal system, which includes the kidneys, is responsible for filtering blood, maintaining fluid and electrolyte balance, and regulating blood pressure [19].

The hepatic system assumes a pivotal role in upholding cardiovascular well-being through the synthesis of essential coagulation proteins, notably fibrinogen and prothrombin. In the absence of these proteins, hemostasis is compromised, culminating in uncontrollable hemorrhage and fatal outcomes [20]. The liver also produces albumin, a protein that helps to maintain fluid balance in the body [21]. Albumin mitigates edema by regulating tissue fluid accumulation, averting swelling, and preserving organ function. Additionally, the liver is responsible for the metabolism of drugs and toxins that may adversely impact the cardiovascular system [22]. When the liver is not functioning properly, these drugs and toxins can accumulate in the body and cause damage to the heart and blood vessels.

The renal system also plays an important role in maintaining cardiovascular health. The renal system is tasked with the filtration of metabolic waste substances from the bloodstream and the elimination of surplus bodily fluids [23]. When the kidneys are not functioning properly, waste products and excess fluid can accumulate in the body, leading to a condition called uremia [24]. Uremia can cause a variety of symptoms, including fatigue, nausea, and shortness of breath. In addition to these symptoms, uremia can also have negative effects on the cardiovascular system [24]. Uremic toxins can cause inflammation in the blood vessels, increasing the risk of developing atherosclerosis, a condition in which plaques build up on the walls of the arteries. Atherosclerosis can increase the risk of heart attack and stroke.

The cardiovascular system, in turn, can have a significant impact on the hepatic and renal systems. The heart is responsible for pumping blood to the liver and kidneys, providing them with the oxygen and nutrients they need to function properly. When the heart is not functioning properly, blood flow to the liver and kidneys can be impaired, which can damage these organs [25].

This study sought to explore the role of *T. gondii* on the cardiovascular, renal, and hepatic systems simultaneously using data from the National Health and Nutrition Examination Survey. The direction through which the damage occurred was unclear; therefore, we explored both directions. The first direction is the association between these chronic diseases and susceptibility to *Toxoplasma* infection. The second direction was the association of *Toxoplasma* in the presence of a chronic disease with the other chronic diseases investigated in this study.

## 2. Materials and Methods

### 2.1. Hypothesis

This study hypothesized that exposure to *Toxoplasma gondii* infection has a negative effect on the cardiovascular system, renal system, and hepatic system simultaneously by causing inflammation, oxidative stress, and tissue damage, leading to an increased risk of cardiovascular disease, chronic kidney disease, and chronic liver disease. To test this hypothesis, we examined the effect of *T. gondii* on an overall cardiovascular biomarker index (OCBI) developed in our prior work [5], a chronic liver disease index, and a chronic kidney disease index used in our prior work [11,13].

### 2.2. Study Population

The National Health and Nutrition Examination Survey (NHANES) is a program conducted by the National Center for Health Statistics (NCHS) of the Centers for Disease Control and Prevention (CDC) in the United States. NHANES collects health and nutrition data from a representative sample of the US population through a complex, multistage probability sampling design. The NHANES 2009–2010 study population consisted of 10,149 individuals, ages 0–85 years, who were selected from households located in counties across the US. However, only 2061 individuals were included in this study who were older than 19 years and had either a *Toxoplasma* infection, chronic cardiovascular disease, CKD, or CLD.

The NHANES 2009–2010 study population was diverse in terms of race/ethnicity, with 70% non-Hispanic Whites, 12% non-Hispanic Blacks, 12% Mexican Americans, and 6% other races/ethnicities. This study population was evenly distributed between males and females. The majority of participants (57%) were aged 20–64 years, while 26% were younger than 20 years, and 17% were 65 years or older.

NHANES 2009–2010 collected a wide range of health and nutrition data from the study population, including medical history, physical examination, laboratory testing, and dietary assessments. The data collected in this study has been used to monitor the health and nutritional status of the US population, as well as to inform public health policies, guide healthcare interventions, and identify trends and disparities in health outcomes.

### 2.3. Study Indicators for Cardiovascular, Kidney, and Liver

In this study, cardiovascular, kidney, and liver health were evaluated using biomarker indexes developed in our previous studies [5,11,13].

#### 2.3.1. Cardiovascular Disease

An overall cardiovascular biomarker index (OCBI) had been developed using eight biomarkers (Table 1). Each biomarker was assigned an index value (0 or 1) based on the threshold recommended clinical practices, giving a value of 0 to the undesirable values. The sum of the index values of all the biomarkers is the OCBI.

#### 2.3.2. CKD

CKD was classified into negative and positive (all stages) based on the calculated estimated glomerular filtration rate (eGFR) and persistent albuminuria.

- Stage 1: eGFR ≥ 90 mL/min/1.73 m^2^ and estimated persistent albuminuria;
- Stage 2: eGFR 60–89 mL/min/1.73 m^2^ and estimated persistent albuminuria;
- Stage 3: eGFR 30–59 mL/min/1.73 m^2^;
- Stage 4: eGFR 15–29 mL/min/1.73 m^2^;
- Stage 5: eGFR < 15 mL/min/1.73 m^2^.

#### 2.3.3. CLD

CLD was categorized in this analysis based on gender and the level of alanine aminotransferase (ALT) or aspartate aminotransferase (AST). Positive CLD was defined as [30]:

- Men: ALT > 40 U/L or AST > 37 U/L
- Women: ALT or AST > 31 U/L

### 2.4. Statistical Analysis

The association of *Toxoplasma* with the combination of cardiovascular disease, CKD, and CLD was conducted using logistic regression models. The holistic assessment was conducted in two directions. The first direction was to evaluate the association of OCBI, CLD, and CKD with *Toxoplasma* infection, where *Toxoplasma* (positive/negative) was used as the outcome. The second direction was to evaluate the association of *Toxoplasma* along with OCBI, CLD, or CKD using three individual models where OCBI, CLD, or CKD was used as the outcome.

The analysis was conducted using R (version 4.0.2; R Foundation for Statistical Computing, Vienna, Austria). To account for the survey weights and sampling design of NHANES, the statistical analysis utilized the “Survey” package in R, which is specifically designed for analyzing complex survey data. A *p*-value < 0.05 was considered significant in all our analyses.

## 3. Results

### 3.1. Study Participants’ Characteristics

Table 2 provides statistical summaries of the variables utilized in this research, including survey-weighted percentages and means. The total number of participants in this sample was 2061, with 42.1% male and 52.8% female. Among these participants, 15.1% were *Toxoplasma*-positive. The mean age was 48.12. The participants included 9.4% Mexican American, 5.5% other Hispanic, 69.8% non-Hispanic white, 9.6% non-Hispanic black, and 5.8% other race. The percentage of CLD was 16.2 and CKD was 10.3, while the median of OCBI-Subindex1 was 5.0.

### 3.2. Holistic Assessment

**Figure 1:**
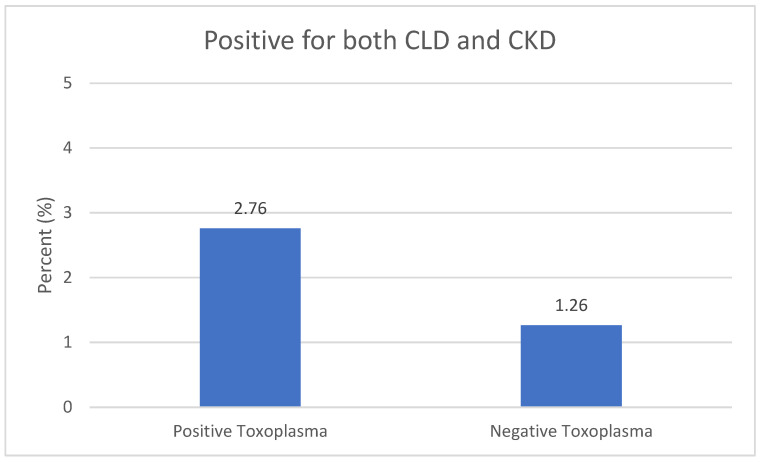
Infection with a combination of CLD and CKD vs. *Toxoplasma* Infection. Chronic liver disease (CLD) and chronic kidney disease (CKD).

Figure 1 shows that the percentage of participants with a combination of CLD and CKD was higher among *Toxoplasma*-positive participants compared to the negative participants (2.76 vs. 1.26).

A holistic assessment was conducted in two directions: the first direction was to evaluate the association of OCBI, CLD, and CKD with *Toxoplasma* infection; the second direction was to evaluate the association of *Toxoplasma* along with OCBI, CLD, or CKD with each of these chronic diseases. Table 3 shows that positive CLD increased the odds of having positive *Toxoplasma* (OR = 1.42, 95% CI = 1.07–1.90, *p* = 0.0149), and OCBI had a negative association with positive *Toxoplasma* (OR = 0.92, 95% CI = 0.85–0.99, *p* = 0.0448). The association of CKD was not apparent in this model; however, adjusting the model for age factor (Table 4) showed that negative CKD decreased the odds of having *Toxoplasma* infection (OR = 0.64, 95% CI = 0.45–0.90, *p* = 0.0119). Also, the positive CLD was associated with *Toxoplasma* infection in this model (OR = 1.72, 95% CI = 1.28–2.31, *p* = 0.0001). The association of OCBI with *Toxoplasma* infection was not apparent in this age-adjusted model.

The first model in the second direction (Table 5) evaluated the association of OCBI, CKD, and *Toxoplasma* infection with CLD after adjusting for age. The model showed that OCBI-Subindex1 was negatively associated with CLD (OR = 0.63, 95% CI = 0.58–0.68, *p* = 0.0001), while *Toxoplasma* infection increased the odds of CLD (OR = 1.68, 95% CI = 1.25–2.26, *p* = 0.0006). Moreover, CKD was not associated with CLD in this model (OR = 0.84, 95% CI = 0.54–1.30, *p* = 0.4445).

The second model in the second direction (Table 6) evaluated the association of OCBI, CLD, and *Toxoplasma* infection with CKD after adjusting for age. The model showed that OCBI was negatively associated with CKD (OR = 0.90, 95% CI = 0.80–0.91, *p* = 0.0353), while *Toxoplasma* infection increased the odds of CKD (OR = 1.50, 95% CI = 1.60–2.13, *p* = 0.0221). However, CLD was not associated with CKD in this model (OR = 1.01, 95% CI = 0.67–1.56, *p* = 0.9557).

The third model in the second direction (Table 7) evaluated the association of CKD, CLD, and *Toxoplasma* infection with OCBI after adjusting for age. The model showed that negative CKD decreased the odds of having low OCBI (OR = 0.20, 95% CI = 0.08–0.50, *p* = 0.0006). While age was significant in this model, *Toxoplasma* was not associated with OCBI in this model. In this holistic evaluation, the model with the highest R square was the CKD model (0.22).

## 4. Discussion

Our results showed that *Toxoplasma* infection was associated with an increased level of cardiovascular biomarkers and lower OCBI, indicating its negative effect on the cardiovascular system. The holistic analysis revealed individuals with higher OCBI had lower odds of *Toxoplasma* (OR = 0.92, 95% CI = 0.85–0.99, *p* = 0.0448), while positive CLD increased the risk of *Toxoplasma* (OR = 1.42, 95% CI = 1.07–1.90, *p* = 0.0149). This study is in concordance with previous research that has consistently elucidated the deleterious consequences of Toxoplasma infection on the cardiovascular and hepatic systems. Multiple investigations have documented an association between Toxoplasma exposure and an elevated risk of cardiovascular disorders, encompassing coronary heart disease and cerebrovascular events, alongside hepatic conditions, notably chronic liver disease. These findings, taken together, underscore the imperative of comprehending and mitigating the plausible health ramifications linked to Toxoplasma infection, especially within the framework of these pivotal physiological organ systems [5,6,13,31,32]. The immune system plays an important role in susceptibility to *Toxoplasma*; however, the cardiovascular system works with the immune system through hormones, cytokines, and neurotransmitters and the health of this system is essential in this connection [33]. The efficiency of this communication is affected by several factors, such as physical and phycological stressors. Degradation of cardiovascular health may unbalance this communication and increase the risk of *Toxoplasma* infection.

The immune system provides three types of immunity: innate, adaptive, and passive immunity [34]. The innate immunity is the natural immunity that a person is born with, and it consists of several elements, including antimicrobial peptides, immune cells, and pattern recognition receptors (PRRs). The liver is the main provider of PRRS in the human body, such as CRP, lipopolysaccharide binding protein, soluble CD14,17–19, peptidoglycan recognition protein, nucleotide binding oligomerization (NOD)-like receptors, RNA helicases, and toll-like receptors [35]. Deterioration of liver function may reduce the production of PRRS, weaken the immune system, and increase the risk of *Toxoplasma* infection. This supports the findings of the holistic analysis, which showed CLD increased the risk of *Toxoplasma* infection.

Studies have highlighted the complex interaction between cardiovascular health and hepatic system health. Several mechanisms were suggested, including inflammation, insulin resistance, endothelial dysfunction, atherosclerosis, and oxidative stress [36]. The holistic analysis showed that cardiovascular disease and *Toxoplasma* infection significantly increased the odds of CLD (OR = 1.68, 95% CI = 1.25–2.26, *p* = 0.0006).

On the other hand, several studies indicated an association between chronic cardiovascular disease and CKD; people with low eGFR and elevated albuminuria had an increased incidence of cardiovascular disease [37]. Furthermore, CKD was suggested to increase the risk of cardiovascular disease more than other risk factors such as diabetes mellitus hypertension [37]. The holistic model in this study supported this finding and showed the association of OCBI and CKD (OR = 0.90, 95% CI = 0.80–0.91, *p* = 0.0353). Furthermore, besides cardiovascular disease, *Toxoplasma* infection increased the odds of CKD in this model (OR = 1.50, 95% CI = 1.60–2.13, *p* = 0.0221). The immune system can also play a role in mediating the association between CKD, cardiovascular disease, and *Toxoplasma*, since the accumulation of uremic toxins such as protein-bound uremic retention solutes during the progression of CKD weakens the innate and adaptive immunity [38]. This was indicated in the association of CKD with *Toxoplasma* infection in the holistic model, where negative CKD decreased the odds of *Toxoplasma* (OR = 0.64, 95% CI = 0.45–0.90, *p* = 0.0119). This corresponds with previous investigations that have demonstrated the adverse impact of *Toxoplasma* on the renal system. Several studies have reported a clear association between *Toxoplasma* exposure and an increased risk of renal disorders, including CKD. These collective findings underscore the significance of comprehending and addressing the potential health implications associated with *Toxoplasma* infection, particularly in the context of the renal system, which plays a critical role in maintaining physiological homeostasis [11,39].

The health of the human system, including the immune system, is essential to minimizing the risk of *Toxoplasma* infection and its health complications. A healthy human body reacts to *T. gondii* infection by activating innate immunity and triggering acquired immune responses, which induce the production of the proinflammatory cytokine interferon-γ (IFN-γ) and activate IFN-γ-inducible proteins to accumulate in parasitophorous vacuole membranes in order to inactivate or inhibit *T. gondii* [40]. Thus, chronic diseases such as CLD and CKD may diminish the immune response to *T. gondii* infection.

Determining the causal relationship between exposure to *T. gondii* and the development of chronic diseases, including CVD, CLD, and CKD, continues to pose a formidable challenge, especially when limited to the confines of cross-sectional data analysis. While the complexity of disentangling causality from correlation in such studies cannot be overstated, emerging evidence underscores the undeniable impact of *T. gondii* on these organ systems. This recognition, in turn, supports a compelling argument that, irrespective of the precise directionality of the association, preventative measures aimed at reducing *T. gondii* infection can significantly mitigate the risk or severity of these debilitating diseases [41,42,43].

### Limitaitons

This study was a cross-sectional study using data gathered at a particular point in time. Because the temporality of association has a strong cause-and-effect criterion, this study cannot demonstrate causation; however, it makes it possible to generate a causal hypothesis of *T. gondii*’s role in chronic diseases, including cardiovascular, kidney, and liver chronic diseases. A future longitudinal study will be ideally suited to confirm the associations identified in this study.

## 5. Conclusions

Toxoplasmosis is an issue of significant public health importance and can contribute to the development of several chronic diseases, including cardiovascular disease, chronic kidney disease, and chronic liver disease. More research is needed to fully understand the relationship between *Toxoplasma gondii* infection and these conditions and to develop effective strategies for preventing and treating toxoplasmosis-related complications. The growing evidence linking toxoplasmosis to chronic diseases underscores the importance of taking steps to prevent exposure to the parasite, particularly for individuals with weakened immune systems or other underlying health conditions. Our findings suggested that exposure to *Toxoplasma gondii* may increase the odds of cardiovascular disease, CKD, or CLD, or vice versa.

This study highlights the implications of parasitic infection on the biomarkers of chronic diseases. In addition, the results of this study have the potential to increase awareness of the health complications of *Toxoplasma* and the need for increased education efforts among the most vulnerable populations and occupations about the silent infection of *Toxoplasma*.

## Figures and Tables

**Table 1:** Overall Cardiovascular Biomarkers Index (OCBI).

Cardiovascular Biomarker	Biomarker Value Assigned Index Value (1)	Biomarker Value Assigned Index Value (0)	Reference
Diastolic blood pressure (DBP)	≤80	>80	[26]
Systolic blood pressure (SBP)	≤120	>120	[26]
High-density lipoprotein (HDL)	≥50 mg/dL	<50 mg/dL	[27]
Triglycerides (TG)	≤199 mg/dL	>199 mg/dL	[26]
C-reactive protein (CRP)	≤1 mg/dL	>1 mg/dL	[28]
Gamma glutamyl transferase (GGT)	≤40 U/L	>40 U/L	[29]
Total cholesterol (TC)	<200 mg/dL	>200 mg/dL	[26]
Glucose (FG)	<100 mg/dL	>100 mg/dL	[26]
Overall Biomarkers Index	Sum of the index values		[26]

**Table 2:** Summary Statistics for the Characteristics of Sample Participants, *n* = 2061.

Variables	Description	*n*	Weighted Percentage/Mean (SE)
*T. gondii* IgG Antibody	<33 IU/mL (IgG−)	1662	85.1
	≥33 IU/mL (IgG+)	399	15.1
Gender	Male	984	47.2
	Female	1077	52.8
Age		2061	48.12 (0.56)
Race/ethnicity	Mexican American	412	9.4
	Other Hispanic	236	5.5
	Non-Hispanic White	1006	69.8
	Non-Hispanic Black	314	9.6
	Other Race	93	5.8
Chronic Liver Disease (CLD)	Negative	1730	83.8
	Positive	331	16.2
Chronic Kidney Disease CKD	Negative	1790	89.7
	Positive	271	10.3
Overall Cardiovascular Biomarker Index (OCBI) median		2061	5.0

**Table 3:** First Direction—Results of Logistic Regression Model for *T. gondii* (IgG+/IgG−) on OCBI- Subindex1, CLD, and CKD.

	*T. gondii* (Positive)			
Sample Size = 2061	Odds Ratio	OR 95% Confidence Interval		*p*-Value
OCBI	0.92	0.85	0.99	0.0448
CLD (positive)	1.42	1.07	1.90	0.0149
CKD (negative)	1.06	0.87	1.46	0.7347
Constant Pseudo R-Square = 0.01	0.37	0.22	0.55	0.0001

Chronic liver disease (CLD), chronic kidney disease (CKD), Overall Cardiovascular Biomarker Index (OCBI).

**Table 4:** First Direction—Results of Logistic Regression Model for *T. gondii* (IgG+/IgG−) on OCBI-Subindex1, CLD, and CKD—Adjusted for Age.

	*T. gondii* (Positive)			
Sample Size = 2061	Odds Ratio	OR 95% Confidence Interval		*p*-Value
OCBI	0.99	0.91	1.08	0.9310
CLD (positive)	1.72	1.28	2.31	0.0001
CKD (negative)	0.64	0.45	0.90	0.0119
Age	1.03	1.02	1.04	0.0001
Constant Pseudo R-Square = 0.03	0.05	0.02	0.10	0.0001

Chronic liver disease (CLD), chronic kidney disease (CKD), Overall Cardiovascular Biomarker Index (OCBI).

**Table 5:** Second Direction—Results of Logistic Regression Model for CLD on *T. gondii* (IgG+/IgG−), OCBI-Subindex1, and CKD—Adjusted for Age.

	CLD			
Sample Size = 2061	Odds Ratio	OR 95% Confidence Interval		*p*-Value
OCBI	0.63	0.58	0.68	0.0001
*Toxoplasma* (positive)	1.68	1.25	2.26	0.0006
CKD (negative)	0.84	0.54	1.30	0.4445
Age	1.07	1.06	1.08	0.0001
Constant Pseudo R-Square = 0.09	7.17	3.81	13.51	0.0001

Chronic liver disease (CLD), chronic kidney disease (CKD), Overall Cardiovascular Biomarker Index (OCBI).

**Table 6:** Second Direction—Results of Logistic Regression Model for CKD on *T. gondii* (IgG+/IgG−), CLD, and OCBI-Subindex1—Adjusted for Age.

	CKD			
Sample Size = 2061	Odds Ratio	OR 95% Confidence Interval		*p*-Value
OCBI	0.90	0.80	0.91	0.0353
CLD (positive)	1.01	0.67	1.56	0.9557
*Toxoplasma* (positive)	1.50	1.60	2.13	0.0221
Age	1.01	1.0	1.02	0.0001
Constant Pseudo R-Square = 0.22	579.59	226.54	1482.81	0.0001

Chronic liver disease (CLD), chronic kidney disease (CKD), Overall Cardiovascular Biomarker Index (OCBI).

**Table 7:** Second Direction—Results of Logistic Regression Model for OCBI-Subindex1 on *T. gondii* (IgG+/IgG−), CLD, and CKD—Adjusted for Age.

	OCBI			
Sample Size = 2061	Odds Ratio	OR 95% Confidence Interval		*p*-Value
CKD (negative)	0.20	0.08	0.50	0.0006
CLD (positive)	1.10	0.51	2.30	0.8117
*Toxoplasma* (positive)	1.70	0.70	4.02	0.2345
Age	1.03	1.0	1.05	0.0225
Constant Pseudo R-Square = 0.06	1.03	0.30	3.48	0.9360

Chronic liver disease (CLD), chronic kidney disease (CKD), Overall Cardiovascular Biomarker Index (OCBI).

## Data Availability

The NHANES dataset is publicly available online, accessible at cdc.gov/nchs/nhanes/index.htm (accessed on 29 September 2023).
